# Association between urinary hCG trajectories and the risk of miscarriage in women undergoing embryo transfer: a prospective cohort study

**DOI:** 10.3389/fendo.2026.1822884

**Published:** 2026-06-04

**Authors:** Jing Wang, Xin Wang, Liming Wang, Xiao Zhang, Pengfei Zang, Wenlong Han, Lu Chen, Qingkai Wang, Yi Li, Liyi Cai

**Affiliations:** 1 Department of Reproduction, Hebei Maternity Hospital, Shijiazhuang, Hebei, China; 2 Shi Jiazhuang Technology Innovation Center of Precision Prevention and Control of Birth Defects, Shijiazhuang, Hebei, China

**Keywords:** early miscarriage, early pregnancy, hCG, IVF-ET, trajectory patterns

## Abstract

**Objective:**

To investigate the association between the dynamic trajectories of urinary human chorionic gonadotropin (hCG) and the risk of early miscarriage after embryo transfer.

**Methods:**

In this prospective cohort study conducted in Hebei Maternity Hospital, 207 women undergoing embryo transfer were enrolled. Starting from the 7th day after transfer, urinary hCG levels were quantitatively measured daily using a latex immunochromatography assay. Early pregnancy development was monitored, with miscarriage as the primary outcome. Latent class trajectory modeling was applied to identify distinct hCG trajectory patterns. Multivariate logistic regression was used to assess the relationship between hCG trajectories and miscarriage risk. The predictive performance of the trajectory model was evaluated using ten-fold cross-validated ROC analysis, decision curve analysis (DCA), and calibration curves.

**Results:**

Urinary hCG was measured over a period of 7 to 51 days. Three distinct hCG trajectories were identified: a Low-Slow group (15.5%), a Low-Steady group (51.2%), and a High-Steady group (33.3%). Compared with the High-Steady trajectory, the Low-Slow trajectory (aOR = 38.11, 95% CI: 26.51-54.78) and the Low-Steady trajectory (aOR = 1.60, 95% CI: 1.14-2.25) were significantly associated with an increased risk of miscarriage. Model 3 predicted miscarriage with an area under the ROC curve (AUC) of 0.857 (95% CI: 0.835–0.879). DCA indicated limited incremental net benefit, while the calibration curve demonstrated good agreement between predicted probabilities and observed outcomes.

**Conclusion:**

A Low-Slow increasing or Low-Steady increasing urinary hCG trajectory after embryo transfer is associated with a higher risk of early miscarriage. Early recognition of these abnormal hCG dynamics may facilitate risk stratification and personalized clinical management.

## 1 Introduction

Infertility has gradually become a global health issue, affecting an estimated 186 million individuals worldwide. To address the reproductive challenges of infertile patients, the application of *in vitro* fertilization-embryo transfer (IVF-ET) has been increasingly adopted (1). Although IVF-ET has improved clinical pregnancy rates, the higher risk of early miscarriage poses new challenges to embryo transfer techniques (2). Notably, 15-25% of all clinically recognized pregnancies end in miscarriage, an event associated with considerable physical and psychological trauma, particularly for women undergoing IVF-ET (3). Therefore, the timely and accurate prediction of early pregnancy outcomes is of paramount importance for guiding clinical management and improving patient care.

Human chorionic gonadotropin (hCG), a glycoprotein hormone secreted by the syncytiotrophoblast, serves as a pivotal biomarker in early gestation. Its secretion begins around 6–7 days post-fertilization, followed by a rapid rise (typically doubling every 1.5–2 days) during the first five weeks. The doubling time prolongs to 3–4 days after the 7th week, with levels peaking around the 10th week before plateauing (4). This dynamic profile closely reflects embryonic viability (5). Consequently, serum β-hCG measurement has been extensively utilized to predict early pregnancy outcomes in both natural conceptions and IVF-ET pregnancies (6). Studies have consistently established its prognostic value: for instance, Frishman et al (7). reported that low initial serum hCG levels in *in vitro* fertilization (IVF) pregnancies are linked to lower ongoing pregnancy rates and a significantly elevated risk of miscarriage. Similarly, Balasičová et al (8). identified serum hCG concentrations on days 14 and 16 post-embryo transfer, as well as their difference, as statistically significant predictors of biochemical pregnancy. Furthermore, evidence suggests that monitoring the dynamic change of hCG offers superior predictive value compared to a single measurement, as highlighted by Ozer et al (9).

However, serum testing is invasive, costly, and impractical for frequent monitoring, and thus cannot capture the continuous trajectories of hCG secretion (10). Study has found that the growth pattern of hCG in maternal serum is similar to that in urine (11). Against this backdrop, urinary hCG detection presents a convenient alternative for serial monitoring, potentially enabling the capture of critical dynamic changes (12). Despite this potential, the value of quantitative urinary hCG trajectories in predicting miscarriage following embryo transfer remains insufficiently explored.

To address this gap, this prospective cohort study aimed to quantitatively measure daily urinary hCG levels from day 7 after embryo transfer, identify distinct longitudinal hCG trajectories, and investigate their association with the risk of clinical miscarriage. This approach seeks to establish a simple and non-invasive method for the early prediction of pregnancy outcomes in IVF-ET, thereby facilitating timely clinical intervention and personalized patient support.

## 2 Methods

### 2.1 Study population

This prospective cohort study recruited female patients undergoing IVF-ET at the Department of Reproduction in Hebei Maternity Hospital from 20 January 2025 to 13 June 2025. A total of 207 participants who underwent frozen embryo transfer were enrolled.

The inclusion criteria were as follows: 1) Age 20–40 years; 2) First-time frozen embryo transfer; 3) Endometrial thickness ≥7 mm on the transfer day. Exclusion criteria included: 1) Concurrent uterine abnormalities such as uterine malformations, endometrial polyps, or submucosal fibroids; 2) Concurrent endocrine disorders (e.g., uncontrolled thyroid dysfunction or diabetes); 3) History of recurrent implantation failure or recurrent spontaneous miscarriage; 4) Severe (stage III–IV) endometriosis; 5) Chromosomal abnormalities in either partner; 6) Post-transfer use of medications affecting hCG levels. All participants signed informed consent forms, and the study protocol was approved by the Hebei Maternity Hospital Ethics Committee.

The primary study outcome was defined as early pregnancy loss occurring within 12 weeks of gestation, including biochemical pregnancy loss and clinical spontaneous miscarriage before 12 weeks of gestation. Ectopic pregnancy was explicitly excluded due to its distinct pathophysiology and clinical course. This definition was adopted to comprehensively capture early pregnancy failure that does not result in a viable ongoing pregnancy, while ensuring etiological homogeneity by excluding pregnancies with abnormal implantation (13).

### 2.2 Urinary hCG measurements

To monitor hCG dynamics, first-morning urine samples were collected daily from participants starting on day 7 through 8 weeks post-embryo transfer. Quantitative hCG measurements were performed using a latex immunochromatography-based detection kit (Shanghai SanteFam Healthcare Technology Co., Ltd.) coupled with a matched automated analyzer. The assay possesses a dynamic detection range of 5 to 5,000 mIU/mL with a limit of detection (LOD) of ≤5 mIU/mL. The analytical reliability of the kit was robust, characterized by high linearity (r ≥ 0.9900), acceptable relative bias (≤ ± 15%), and intra- and inter-assay coefficients of variation (CV) of ≤10.0% and ≤15.0%, respectively.

**Figure 1:**
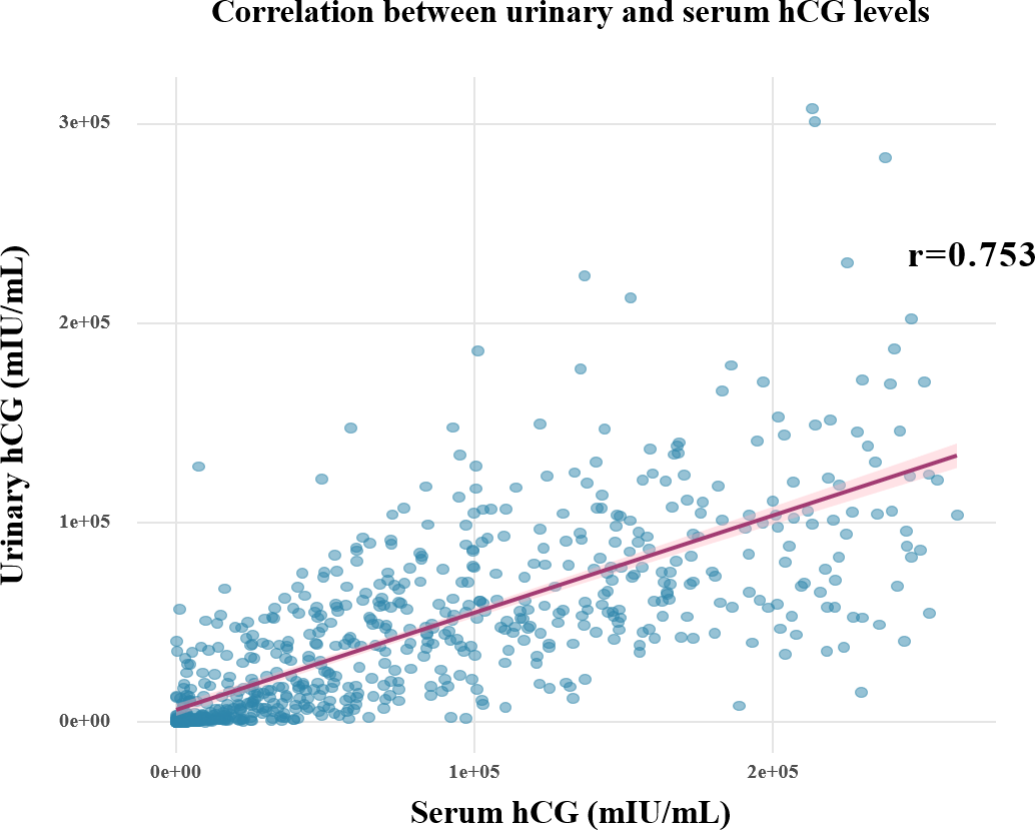
Correlation analysis between urinary and serum hCG.

To evaluate the consistency between urinary hCG testing and serum hCG testing, this study simultaneously collected venous blood samples at corresponding time points and determined serum hCG concentrations using chemiluminescence assay in the hospital laboratory. Pearson correlation analysis comparing urinary hCG and serum hCG results at the same time point revealed a high correlation (r = 0.753, P<0.001), indicating that quantitative urinary hCG testing can effectively reflect serum hCG levels under these study conditions and is feasible as a substitute for blood testing (**Figure 1**).

### 2.3 Assessment of covariates

Data were collected through structured questionnaires and medical records. Sociodemographic characteristics included age (recorded in years), body mass index (BMI, calculated as weight in kilograms divided by height in meters squared), and self-reported menstrual regularity. Given that the study population consisted of Chinese participants, menstrual cycle regularity was defined according to criteria commonly applied in Chinese clinical practice (14): cycles lasting 21–35 days were categorized as regular, and those outside this range as irregular. Embryo transfer characteristics included the number of embryos transferred (single or double) and the type of transferred embryo (blastocyst or cleavage-stage embryo). These variables were selected based on their known or potential influence on hCG levels and early pregnancy outcomes. Although the selected covariates were limited, they represent the most commonly reported and biologically relevant predictors in early reproductive research.

### 2.4 Statistical analysis

Baseline was defined as the time of embryo transfer, with data collected via questionnaires. Baseline characteristics were presented as mean ± standard deviation (SD) for continuous variables and as frequencies (percentages) for categorical variables. Differences in population characteristics across the identified human chorionic gonadotropin (hCG) trajectory groups were compared using one-way ANOVA or Chi-square tests, as appropriate.

To account for variations in urine concentration, raw longitudinal urinary hCG measurements were first standardized by adjusting for mean urinary creatinine levels and urine specific gravity, calculated using the following formula:


$$\text{hCG}\left(\text{adjusted}\right)=\text{hCG}\left(\text{measured}\right)\times \frac{\text{Creatinine}\left(\text{actual}\right)}{\text{Creatinine}\left(\text{mean}\right)}\times \text{f}\left(\text{urine specific gravity}\right)$$

Given the exponential growth characteristics of early hCG secretion, the standardized hCG values subsequently underwent logarithmic transformation. These log-transformed data were then utilized to construct accurate growth trajectory models via latent class trajectory modeling (LCTM). The optimal number of latent classes was determined based on the lowest Akaike Information Criterion (AIC) and Bayesian Information Criterion (BIC), alongside a high entropy value. Furthermore, a sensitivity analysis was conducted to confirm the robustness of the trajectory classification.

To evaluate the clinical implications of these trajectories, multivariable logistic regression models were constructed to estimate the adjusted odds ratios (aOR) for the risk of miscarriage, adjusting for maternal age, BMI, embryo type, and the number of transferred embryos.

Finally, the predictive performance of the miscarriage risk prediction model was comprehensively assessed Discriminative ability was quantified using the area under the receiver operating characteristic curve (AUC) derived from a 10-fold cross-validation (10-FCV) approach. Calibration was evaluated via calibration curves (both apparent and bias-corrected using 1000 bootstrap resamples) to determine the agreement between predicted and observed probabilities, quantified by the mean absolute error (MAE). Clinical net benefit was assessed using decision curve analysis (DCA) across a spectrum of threshold probabilities. All analyses were performed using R version 4.3.3. Statistical tests were two-sided, with a significance level set at p< 0.05 (15).

## 3 Results

### 3.1 Baseline characteristics of the participants

**Figure 2:**
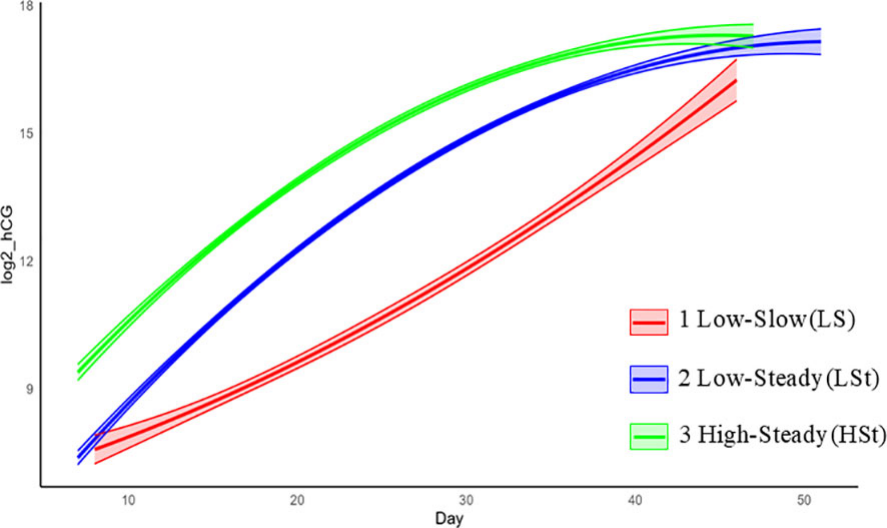
Trajectories in assisted reproductive technology pregnant women.

Through group-based trajectory modeling, we identified three distinct trajectories of urinary hCG among 207 women undergoing embryo transfer. The model with three trajectories showed the best fit to the data (**Supplementary Table 1**). **Figure 2** shows the estimated mean urinary hCG in three trajectories: 15.5% (32) of women maintained an initial low hCG levels, which was slowly increase (Low-Slow group); 51.2% (106) of women started with a low hCG and then increased steady (Low-Steady group); 33.3% (69) of women initially have a high hCG levels, then increased steady(High-Steady group). To better assess the validity and clinical interpretability of the latent class trajectories, we adopted semi-transparent individual trajectories overlaid with the class-specific mean curves (**Supplementary Figure 1**). The baseline characteristics of study participants according to urinary hCG trajectories are shown in **Table 1**. In general, participants from Low-Slow and Low-Steady trajectories were more likely to be older and to have embryos of the cleavage-stage type transferred. The raw hCG values on different days after transfer are shown in **Supplementary Table 2**.

**Table 1:** Baseline characteristics of participants with IVF-ET stratified by trajectories of hCG.

Characteristic	Total	hCG trajectory group			p-value
		Low-Slow	Low-Steady	High-Steady	
No. of participants	207	32	106	69	
Age	32.8 (5)	34.7 (4.1)	32.6 (4.9)	32.3 (4.5)	0.045
Age_cat					0.091
<35	124 (60%)	14 (44%)	64 (60%)	46 (67%)	
≥35	83 (40%)	18 (56%)	42 (40%)	23 (33%)	
BMI	25.6 (5.2)	26.1 (5.0)	25.9 (5.6)	24.9 (4.6)	0.400
BMI_cat					0.300
<18.5	5 (2.4%)	1 (3.1%)	4 (3.8%)	0 (0%)	
18.5∼24	86 (42%)	10 (31%)	44 (42%)	32 (46%)	
24∼28	58 (28%)	11 (34%)	25 (24%)	22 (32%)	
≥28	58 (28%)	10 (31%)	33 (31%)	15 (22%)	
Regularity of menstruation					0.900
Yes	132(64%)	27(63%)	67(63%)	45(65%)	
No	75(36%)	12(38%)	39(37%)	24(35%)	
Embryo type					<0.001
Cleavage	72 (35%)	17 (53%)	46 (43%)	9 (13%)	
Blastocyst	135 (65%)	15 (47%)	60 (57%)	60 (87%)	
Number of embryos					0.072
1	146 (71%)	27 (84%)	76 (72%)	43 (62%)	
2	61 (29%)	5 (16%)	30 (28%)	26 (38%)	

### 3.2 Associations of urinary hCG trajectories with miscarriage

**Figure 3:**
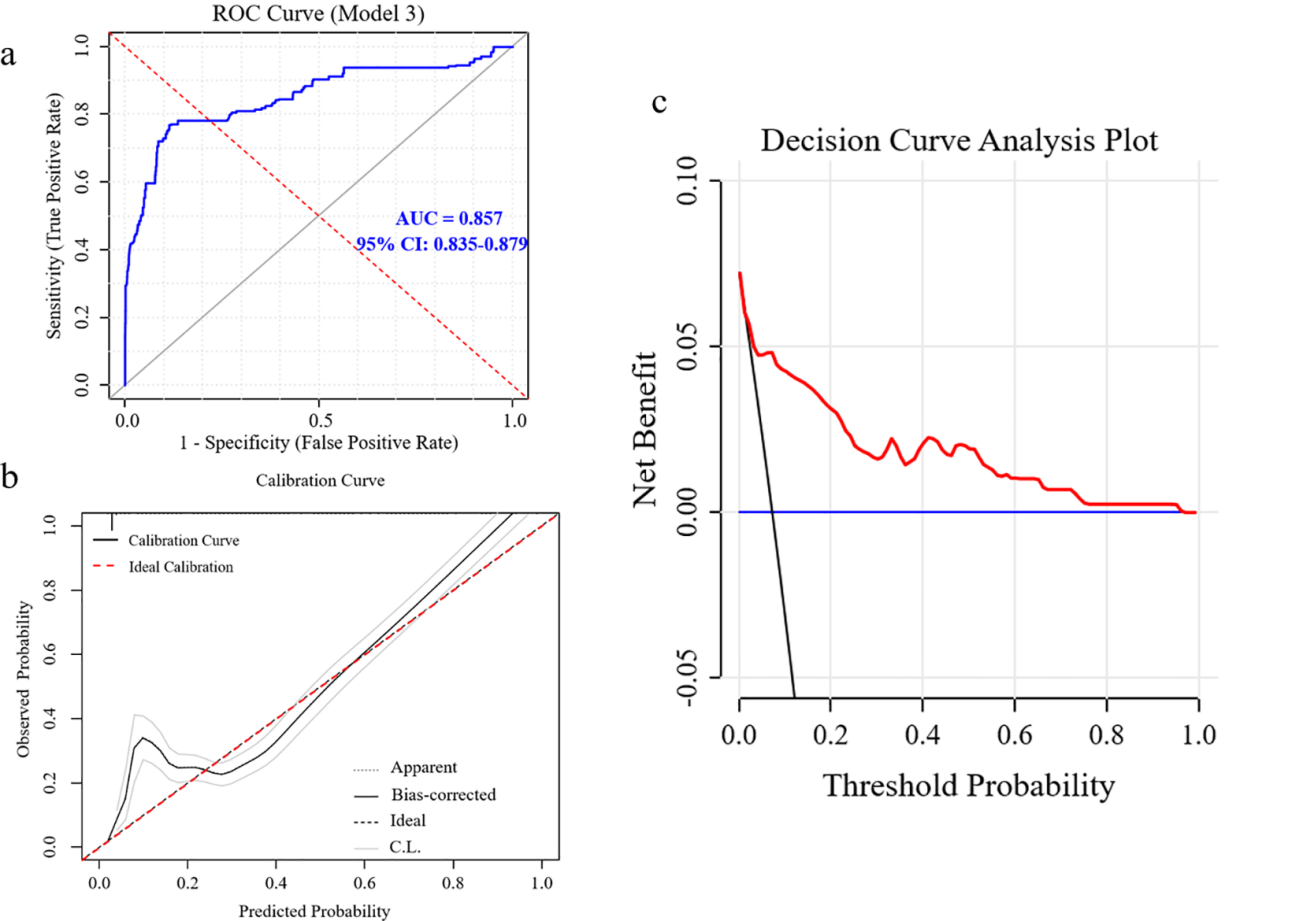
Comprehensive performance assessment of hCG trajectories [**(a)** ROC curve, **(b)** Calibration curve, **(c)** DCA plot].

Compared to women with the “High-Steady” trajectory, those with the “Low-Slow” trajectory (aOR=38.11, 95%CI:26.51-54.78) and the “Low-Steady” trajectory (aOR=1.60, 95%CI:1.14-2.25) had an increased risk of miscarriage (**Table 2**). Meanwhile, we subsequently developed a prediction model for early miscarriage, incorporating the identified hCG trajectories as a key predictor alongside other clinical covariates (Model 3). The urine hCG trajectory model shows a good discriminatory ability in predicting the risk of miscarriage in women undergoing IVF-ET. The average AUC of 10-fold cross-validation was 0.857 (95%CI: 0.835-0.879). To further validate the selected model, a sensitivity analysis comparing the 2-class, 3-class, and 4-class trajectory models was performed. The analysis revealed that while the overarching directional trends of the hCG trajectories remained highly consistent across the different classifications, the 3-class model achieved the most optimal discriminative performance (2-class AUC = 0.831,95%CI:0.809-0.853; 4-class AUC = 0.785,95%CI:0.761-0.81) for predicting early miscarriage (**Supplementary Figure 2**), thereby confirming its appropriateness for our primary analysis. Calibration curve analysis shows that the predicted probability of miscarriage is highly consistent with the observed values. The calibration curve after bias correction coincides highly with the ideal calibration line. The DCA results show that the net benefit curve of the model is consistently higher than the baseline curve throughout the entire miscarriage threshold probability range (0 to 1) (**Figure 3**).

**Table 2:** Association of hCG trajectories and pregnancy.

hCG trajectory	Participants	cases	Model 1	Model 2	Model 3
High-steady	69	2	1.00	1.00	1.00
Low-slow	32	17	26.59(19.63-36.01)	29.71(21.61-40.83)	38.11(26.51-54.78)
Low-steady	106	5	1.35(0.97-1.87)	1.43(1.03-1.98)	1.60(1.14-2.25)

Model 1 was a crude model; Model 2 adjusted for age and BMI at baseline. Model 3 adjusted for variables in model 2 and the number and types of embryos.

## 4 Discussion

This study demonstrates that the longitudinal trajectory of quantitatively measured urinary hCG serves as a predictor of early miscarriage risk following embryo transfer. By modeling daily levels, we identified three distinct secretion patterns—High-Steady, Low-Steady, and Low-Slow—that were associated with miscarriage. This finding underscores that the dynamic pattern of hCG secretion, encompassing both its initial magnitude and subsequent growth trajectory, provides superior prognostic information compared to a single, static measurement.

Serum hCG detection has long been recognized as a core indicator for evaluating early embryonic viability and has become a clinical consensus in miscarriage risk assessment (6). Previous studies firmly established the prognostic value of serum hCG levels (2, 3, 8, 16). However, the invasiveness of serum testing, significant individual heterogeneity, and high cost make frequent sampling for establishing dynamic growth curves impractical. Quantitative urine hCG testing provides a practical and patient-friendly alternative (12). First, this method accurately measures urinary hCG concentrations, which exhibits a high correlation with serum levels (r=0.753), thereby reliably reflecting trophoblast secretory function and embryonic development. Second, continuous quantitative monitoring enables the detection of early abnormal fluctuations. For example, in this study, some pregnant women with normal baseline urinary hCG levels but slow growth in subsequent quantitative monitoring were eventually diagnosed with missed abortion, which could not be identified by qualitative detection. Third, urinary hCG quantitative detection can provide continuous numerical data, laying the foundation for subsequent dynamic trajectory analysis. Compared with qualitative detection’s “binary outcome” (positive/negative), quantitative data contains richer information, which can more comprehensively characterize the dynamic process of embryonic development and improve the accuracy of risk prediction. Finally, urinary hCG quantitative detection avoids the interference of serum matrix effects (such as protein binding and hormone cross-reaction) and has better stability in repeated measurements, which is conducive to long-term dynamic monitoring.

Our study identified three distinct urinary hCG growth patterns in women undergoing IVF-ET as Low-Slow, Low-Steady, and High-Steady. Specifically, compared with women in the High-Steady group (high initial hCG level with subsequent steady growth), those in the Low-Steady group (low initial hCG level with subsequent steady growth) had an increased risk of miscarriage. More notably, the miscarriage risk was further significantly elevated in the Low-Slow group (low initial hCG level with subsequent slow growth). These findings highlight that both the initial hCG concentration and its subsequent dynamic trajectory are critical for predicting pregnancy outcomes, with the latter potentially playing a more decisive prognostic role in early pregnancy success. From a physiological perspective, initial hCG levels in early pregnancy primarily reflect blastocyst implantation efficiency and the initial functional mass of trophoblast cells (16). As a glycoprotein hormone secreted by syncytiotrophoblast cells, hCG can be detected in maternal circulation shortly after implantation, and its initial concentration correlates closely with the number and activity of these implanting cells (3). Previous studies have confirmed that higher initial β-hCG levels are correlated with better pregnancy outcomes, including higher clinical pregnancy rates and lower abortion rates, which is consistent with the finding in this study that low initial hCG levels are associated with increased miscarriage risk (17, 18). In contrast, the subsequent growth pattern of hCG is more likely to reflect the continuous functional status of trophoblasts and the overall developmental potential of the embryo (19, 20). Trophoblast cell fusion and continuous proliferation are essential for maintaining normal placental development and sustaining pregnancy, and hCG secretion is tightly regulated by the functional state of trophoblasts (21, 22). Studies have shown that factors promoting trophoblast fusion (e.g., human leukocyte antigen G) can significantly enhance hCG production through regulating the Erk1/2 pathway, while impaired trophoblast function leads to insufficient hCG synthesis and slow growth (21). Therefore, the slow growth of hCG in the Low-Slow group observed in this study may indicate inadequate trophoblast proliferation and fusion, thereby failing to establish effective maternal-fetal circulation and ultimately increasing the risk of miscarriage. However, the notably large effect size observed in the Low-Slow group should be interpreted with caution, as it may reflect potential model instability associated with subgroup sample size limitations or residual confounding. Although this finding suggests a possible strong association, further studies with larger sample sizes and external validation are required to confirm its robustness and clarify the underlying mechanisms of this association.

Based on the urine hCG trajectory, we incorporated female age, baseline BMI, embryo implantation type, and number for comprehensive evaluation of the predictive performance of the hCG trajectory using a 10-fold ROC curve, DCA, and calibration curve. The ROC curve showed that the AUC was 0.857 (95% CI: 0.835-0.879), indicating that the model had excellent discriminative ability to distinguish pregnant women with high and low miscarriage risk. Importantly, the robustness of our model selection was corroborated by the sensitivity analysis. The calibration curve demonstrated a good agreement between the predicted probability of miscarriage and the actual observed miscarriage rate, with the bias-corrected curve close to the ideal calibration line, suggesting reliable calibration of the model. DCA demonstrated that the model provided positive net benefits across all threshold probabilities, suggesting its potential clinical relevance, generating new hypotheses for miscarriage risk intervention, and offering a theoretical basis for future validation studies. The true value of urine hCG trajectories may lie in enabling personalized monitoring and early counseling. For instance, patients identified with a High-Steady trajectory could be reassured, potentially reducing anxiety and unnecessary early visits. Those in higher-risk trajectories could be prioritized for closer surveillance, early physiological luteal support evaluation, or timely psychological intervention, aligning with the goal of personalized reproductive medicine. Due to limitations in data availability, we were unable to incorporate several key factors that are known to influence miscarriage risk in IVF. The omission of these critical variables may introduce residual confounding and affect the overall robustness of our results. Consequently, the clinical implications drawn from this study should be viewed as hypothesis-generating. Future studies with more comprehensive data collection are essential to account for these unmeasured factors and further validate our observations.

The strength of this study lies in the continuous measurement of urinary hCG and the modeling of growth trajectories. In addition, urine sampling is more convenient for home use compared to blood sampling, which may enhance compliance and scalability in clinical practice. This study focuses on embryo transfers in clearly high-risk populations, enhancing the direct relevance of our findings to assisted reproductive care.

Despite these promising findings, several limitations must be acknowledged. First, the relatively small overall sample size constitutes a primary limitation of this study. Although 10-fold cross-validation (10-FCV) was rigorously employed to evaluate model performance and mitigate overfitting, relying solely on this internal validation technique may not fully resolve the potential mathematical instability inherent in limited datasets. This instability is particularly evident regarding the extraordinary effect size observed in the Low-Slow group (aOR = 38.11), which warrants cautious interpretation. Given the small number of target events in this specific subgroup (n = 17), the multivariable statistical model is highly susceptible to data sparsity, class separation, or artificially inflated estimates. Consequently, these particular findings should be viewed as hypothesis-generating rather than definitive, pending rigorous external validation in larger, independent cohorts. Second, owing to data availability constraints, our multivariable model did not adjust for several unmeasured confounders, such as embryonic chromosomal status, subtle immunological markers, or lifestyle stressors, which may introduce residual confounding. Third, as a single-center study, the generalizability of our findings requires confirmation through multicenter validation. Although 10-FCV was employed to mitigate overfitting, independent external validation is imperative before widespread clinical implementation. Finally, while trajectory modeling provides useful risk stratification at the group level, its application for individualized prediction should be integrated with additional clinical and biomarker information.

## 5 Conclusion

In conclusion, this study confirms that urinary hCG quantitative detection combined with latent trajectory models has significant advantages in early identification of miscarriage risk. This method can not only realize early risk stratification of pregnant women but also provide a basis for personalized clinical intervention (such as close monitoring, progesterone supplementation, or psychological counseling), which is of great significance for reducing miscarriage rates and improving pregnancy outcomes.

## Data Availability

The original contributions presented in the study are included in the article/**Supplementary Material**. Further inquiries can be directed to the corresponding authors.
