# Supplementary material for: Association between urinary hCG trajectories and the risk of miscarriage in women undergoing embryo transfer: a prospective cohort study
Source: Front Endocrinol (Lausanne). 2026 Jun 4;17:1822884. doi: 10.3389/fendo.2026.1822884 (PMC13275385; doi:10.3389/fendo.2026.1822884)
Supplement: Supplementary file 1 [file DataSheet1.docx]

**Supplementary information**

Table S**1**: Fit indices and group sizes of the Latent Class Trajectory Models

| Indices | **Unconditional model** | | | |
| --- | --- | --- | --- | --- |
|  | **1-profile** | **2-profile** | **3-profile** | **4-profile** |
| Fit statistics |  |  |  |  |
| AIC | 25534.57 | 23674.86 | **22826.55** | 22500.54 |
| BIC | 25547.9 | 23701.52 | **22866.54** | 22553.87 |
| Entropy | — | 0.979 | **0.947** | 0.950 |
| Group sizes (%) |  |  |  |  |
| C1 | 207 (100%) | 58 (28.01%) | **32 (15.46%)** | 14 (6.76%) |
| C2 | — | 149 (71.98%) | **106 (51.21%)** | 108 (52.18%) |
| C3 | — | — | **69 (33.33%)** | 29 (14.01%) |
| C4 | — | — | — | 56 (27.05%) |

Table **S2**: Distribution of original hCG values

| Day | n | Mean | SD | *P*_25_ | *P*_50_ | *P*_75_ |
| --- | --- | --- | --- | --- | --- | --- |
| 7 | 9 | 896.68 | 586.49 | 579.24 | 687.32 | 1514.4 |
| 8 | 121 | 5010.86 | 11806.73 | 521.64 | 1122.63 | 2250.96 |
| 9 | 133 | 2635.76 | 5705.89 | 246.25 | 838.24 | 1910.7 |
| 10 | 136 | 3398.73 | 8207.02 | 291.61 | 803.8 | 1962.7 |
| 11 | 139 | 2646.9 | 5227.86 | 508.4 | 930.91 | 1767.82 |
| 12 | 151 | 3761.72 | 8108.48 | 573 | 1080.51 | 2490.37 |
| 13 | 202 | 3964.94 | 11007.42 | 531.42 | 1043.13 | 2320.83 |
| 14 | 206 | 3273.19 | 7870.62 | 655.63 | 1176.8 | 2108.71 |
| 15 | 199 | 3907.96 | 9911.5 | 795.96 | 1239.92 | 1939.92 |
| 16 | 199 | 3194.3 | 6853.79 | 768.34 | 1274.87 | 2284.5 |
| 17 | 200 | 3624.42 | 11239.58 | 947.59 | 1443.48 | 2393.12 |
| 18 | 201 | 6243.71 | 10814.59 | 1067.18 | 1725 | 5091 |
| 19 | 201 | 8173.22 | 14380.57 | 1132.31 | 1843.59 | 9110.25 |
| 20 | 202 | 10157.62 | 15894.97 | 1380.86 | 3291.18 | 12892.31 |
| 21 | 195 | 14796.97 | 20468.65 | 1747.4 | 5824.5 | 19292.62 |
| 22 | 195 | 18798.51 | 21260.98 | 2426.56 | 10448.25 | 30768.38 |
| 23 | 196 | 23528.01 | 23622.69 | 5338.12 | 14472 | 38285.81 |
| 24 | 196 | 28252.77 | 26855.97 | 6995.25 | 20238.75 | 42443.62 |
| 25 | 195 | 32695.13 | 27436.45 | 10399.88 | 27589.5 | 47125.12 |
| 26 | 194 | 40443.86 | 34191.69 | 11684.81 | 37900.5 | 56289.75 |
| 27 | 185 | 43553.48 | 32150.66 | 15937.5 | 39327 | 61325.25 |
| 28 | 188 | 49283.03 | 36091.85 | 17392.12 | 45137.25 | 70468.5 |
| 29 | 183 | 58642.84 | 49551 | 27310.88 | 49365 | 80622.75 |
| 30 | 185 | 63358.76 | 59280.71 | 37056 | 51570 | 78129 |
| 31 | 184 | 64729.22 | 45440.22 | 39100.12 | 54846 | 90578.81 |
| 32 | 185 | 67946.95 | 52995.44 | 38525.25 | 57912 | 91605 |
| 33 | 184 | 69849.5 | 44942.58 | 39537.75 | 60849.75 | 95740.5 |
| 34 | 183 | 89801.62 | 105228.89 | 43637.62 | 66267.75 | 105057.75 |
| 35 | 178 | 88486.31 | 90437.44 | 45570.19 | 64965.38 | 107336.62 |
| 36 | 177 | 97934.34 | 96806.92 | 55175.25 | 79420.5 | 112430.5 |
| 37 | 173 | 91052.89 | 76808.92 | 52749.75 | 78888.75 | 108858 |
| 38 | 172 | 113882.66 | 105465.55 | 56612.62 | 92836.12 | 127916.44 |
| 39 | 172 | 109752.65 | 80585.65 | 59223.56 | 92111.25 | 131378.06 |
| 40 | 164 | 118889.95 | 109453.04 | 60817.5 | 96907.12 | 136390.69 |
| 41 | 147 | 112581.17 | 87926.53 | 57997.12 | 96080.25 | 129460.5 |
| 42 | 128 | 111525.3 | 91599.31 | 57762.38 | 89448.75 | 137539.31 |
| 43 | 41 | 103973.74 | 72055.33 | 67329.75 | 91937.25 | 122314.5 |
| 44 | 20 | 79073.25 | 42168.53 | 59281.5 | 83878.88 | 103305.94 |
| 45 | 13 | 80406.92 | 37012.95 | 48518.25 | 80346 | 93824.25 |
| 46 | 9 | 81860.42 | 46644.12 | 54286.5 | 85092.75 | 99105 |
| 47 | 4 | 114648.75 | 28621.02 | 93021.56 | 116658.38 | 138285.56 |
| 48 | 2 | 96803.25 | 52684.05 | 78176.62 | 96803.25 | 115429.88 |
| 49 | 1 | 176546.25 | NA | 176546.25 | 176546.25 | 176546.25 |
| 50 | 1 | 77961.75 | NA | 77961.75 | 77961.75 | 77961.75 |
| 51 | 1 | 52683 | NA | 52683 | 52683 | 52683 |


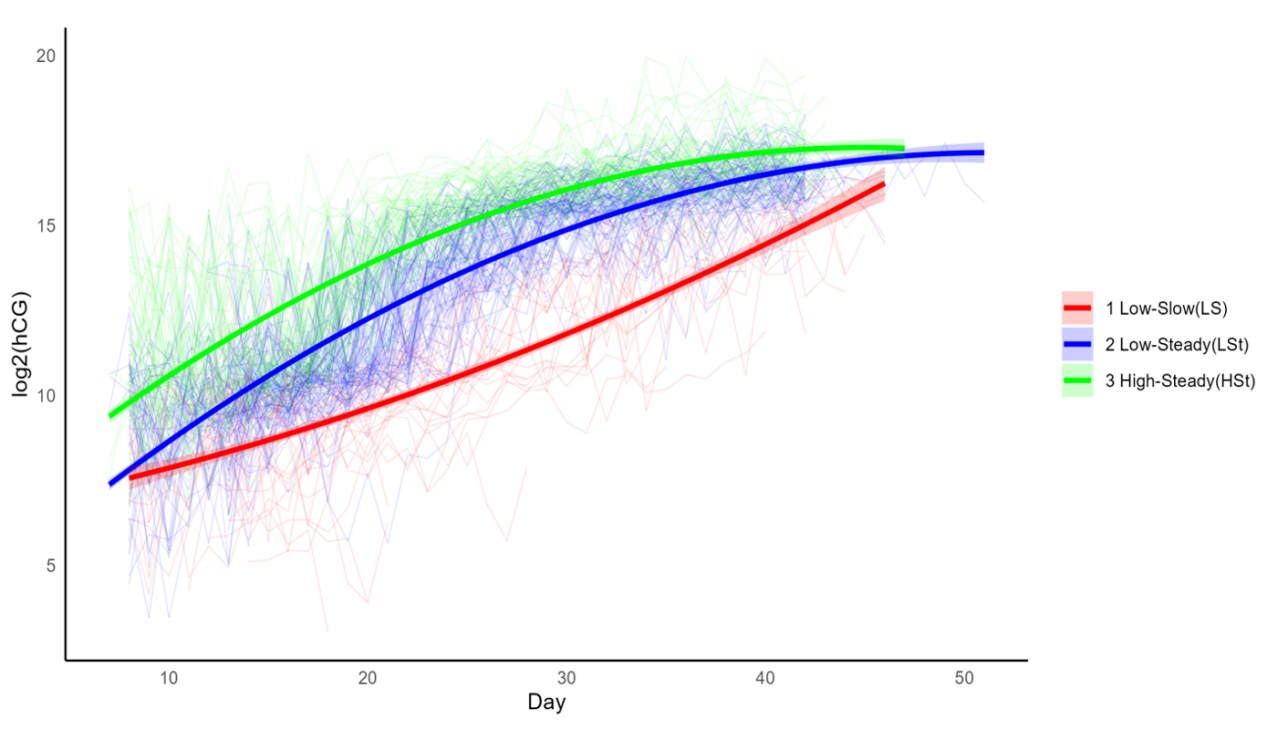


Figure **S1** Individual-level longitudinal trajectories of urinary hCG by latent class, with semi-transparent spaghetti lines showing within-class variability and bold curves representing class-specific predicted means.

| 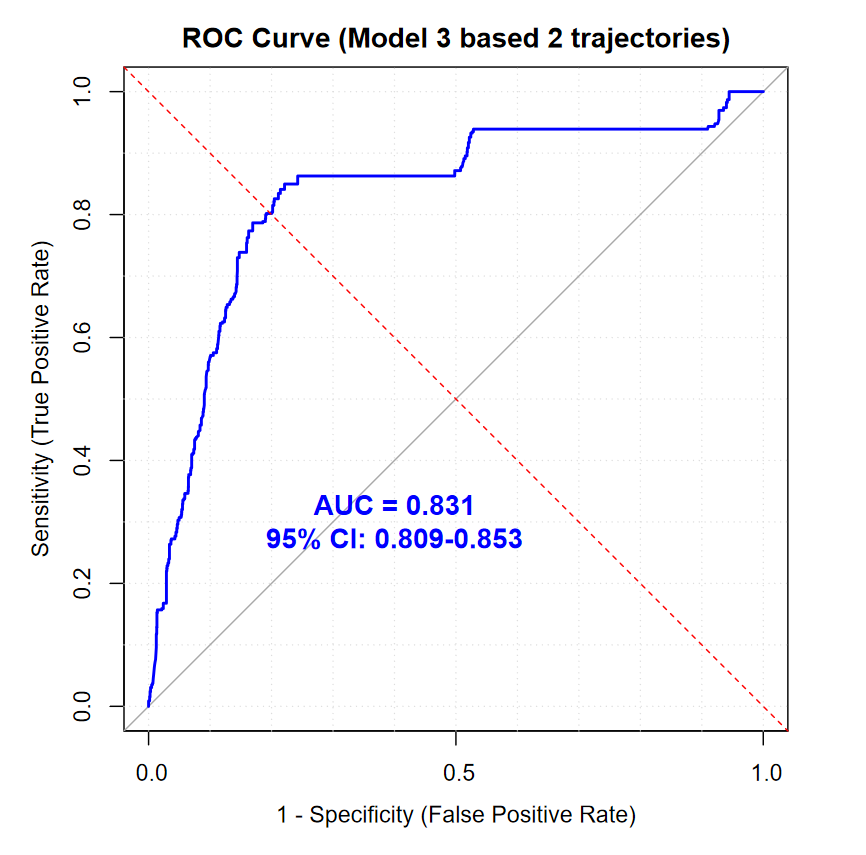 | 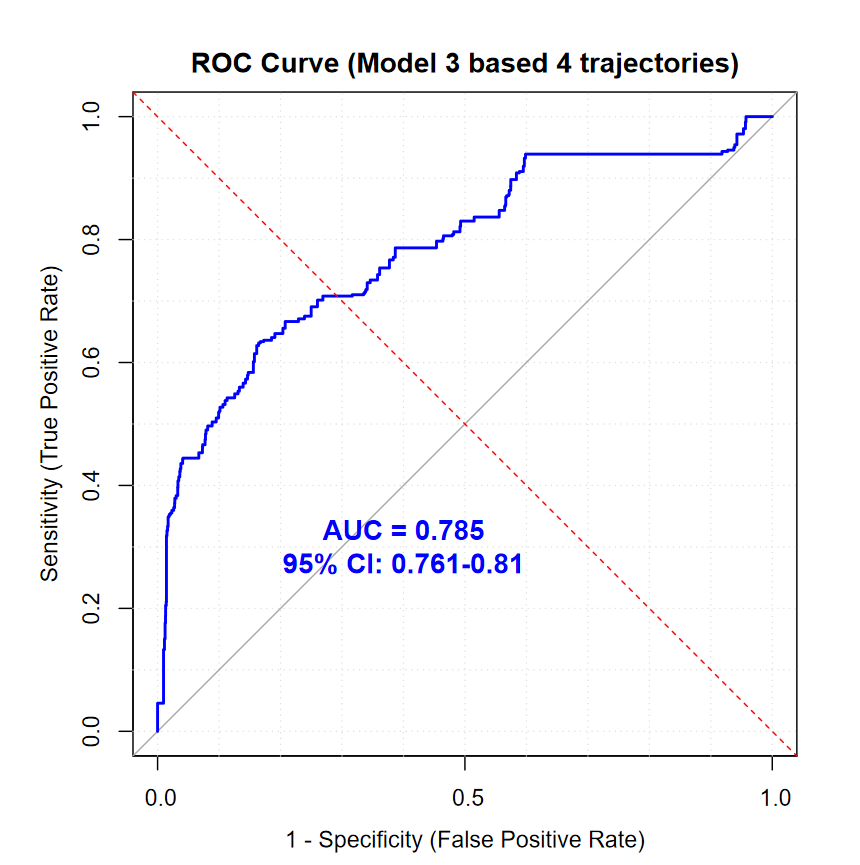 |
| --- | --- |
| A:ROC Curves of 10-fold cross-validation based 2 trajectories | B:ROC Curves of 10-fold cross-validation based 4 trajectories |
|  |  |

Figure **S2** ROC Curves of Cross-validation with Tenfold Split for Different Trajectory Models
